# Nexfin Noninvasive Continuous Hemodynamic Monitoring: Validation against Continuous Pulse Contour and Intermittent Transpulmonary Thermodilution Derived Cardiac Output in Critically Ill Patients

**DOI:** 10.1155/2013/519080

**Published:** 2013-11-11

**Authors:** Koen Ameloot, Katrijn Van De Vijver, Ole Broch, Niels Van Regenmortel, Inneke De laet, Karen Schoonheydt, Hilde Dits, Berthold Bein, Manu L. N. G. Malbrain

**Affiliations:** ^1^Department of Intensive Care Medicine, Ziekenhuis Netwerk Antwerpen, ZNA Stuivenberg, Lange Beeldekensstraat 267, 2060 Antwerp, Belgium; ^2^Department of Anaesthesiology and Intensive Care Medicine, University Hospital Schleswig-Holstein, Campus Kiel, Schwanenweg 21, 24105 Kiel, Germany

## Abstract

*Introduction*. Nexfin (Bmeye, Amsterdam, Netherlands) is a noninvasive cardiac output (CO) monitor based on finger arterial pulse contour analysis. The aim of this study was to validate Nexfin CO (NexCO) against thermodilution (TDCO) and pulse contour CO (CCO) by PiCCO (Pulsion Medical Systems, Munich, Germany). *Patients and Methods*. In a mix of critically ill patients (*n* = 45), NexCO and CCO were measured continuously and recorded at 2-hour intervals during the 8-hour study period. TDCO was measured at 0–4–8 hrs. *Results*. NexCO showed a moderate to good (significant) correlation with TDCO (*R* ^2^ 0.68, *P* < 0.001) and CCO (*R* ^2^ 0.71, *P* < 0.001). Bland and Altman analysis comparing NexCO with TDCO revealed a bias (± limits of agreement, LA) of 0.4 ± 2.32 L/min (with 36% error) while analysis comparing NexCO with CCO showed a bias (±LA) of 0.2 ± 2.32 L/min (37% error). NexCO is able to follow changes in TDCO and CCO during the same time interval (level of concordance 89.3% and 81%). Finally, polar plot analysis showed that trending capabilities were acceptable when changes in NexCO (ΔNexCO) were compared to ΔTDCO and ΔCCO (resp., 89% and 88.9% of changes were within the level of 10% limits of agreement). *Conclusion*. we found a moderate to good correlation between CO measurements obtained with Nexfin and PiCCO.

## 1. Introduction

The true value of continuous hemodynamic monitoring in critically ill patients becomes clear in the light of beat-to-beat changing hemodynamics due to either continuously improving or deteriorating cardiac and disease status [1, 2]. Therefore, the critically ill patient must be resuscitated to a continuously changing optimal left ventricular end-diastolic volume. This should be titrated together with an accurate dose of vasopressor agents and inotropes to optimize circulation and restore end-organ perfusion without causing harm due to excessive fluids [3]. Also, for further decision making, we often want to measure in real-time the hemodynamic effects of ongoing therapeutic interventions. The PiCCO system (PiCCO2, Pulsion Medical Systems, Munich, Germany) uses a dedicated PiCCO thermistor-tipped arterial catheter to analyze the patient's heart rate and arterial pressure waveform continuously [4]. Due to the unique properties of each patient's arterial tree, initial calibration of the monitoring system using transpulmonary thermodilution CO measurement (TDCO) improves accuracy of the beat-to-beat cardiac output (CO) obtained by pulse contour analysis (CCO) [5]. The PiCCO device has been validated in numerous studies including burns, medical, and surgical critically ill patients [6–12]. However, PiCCO remains a relative invasive technique that requires both an arterial and a central venous catheter and therefore increases the risk of iatrogenic complications such as pneumothorax, bleeding, catheter sepsis, and deep venous thrombosis [13–15]. Moreover, the need for calibration by transpulmonary thermodilution may delay the initial measurement and is time consuming, and the system is cost intensive and cannot be used prehospital or in a regular ward. The Nexfin (BMEYE, Amsterdam, The Netherlands) device is a totally noninvasive continuous blood pressure and CO (NexCO) monitor based on finger arterial pressure pulse contour analysis. Potential advantages include its noninvasiveness and ease of use. Questions rose on the accuracy of Nexfin measurements in critically ill patients, as there is no initial calibration of the monitoring system to adjust for the unique mechanical properties of each patient's arterial tree. Moreover, there are concerns about the reliability of continuous noninvasive finger blood pressure derived pulse contour analysis in patients with reduced perfusion of the hand due to high systemic vascular resistance (SVR) or hypothermia. We performed an open observational study in a mix of medical/surgical and burns critically ill patients to validate Nexfin against both TDCO and CCO obtained by PiCCO.

## 2. Methods

### 2.1. Study Population

We prospectively studied 47 critically ill patients admitted to the intensive care units of our hospital. Inclusion criteria were hemodynamic instability with need for continuous hemodynamic monitoring and the presence of a central venous (jugular or subclavian) catheter and a PiCCO femoral arterial catheter already in place before inclusion in the study.

### 2.2. Nexfin Technique

The Nexfin (BMEYE, Amsterdam, The Netherlands) method is based on the measurement of finger arterial pressure by an inflatable cuff around the middle phalange of the finger. The pulsating finger artery is clamped to a constant volume by applying a varying counter pressure equivalent to the arterial pressure using a built-in photoelectric plethysmograph and an automatic algorithm (Physiocal). The resulting finger arterial pressure waveform is reconstructed into a brachial artery pressure waveform by a generalized algorithm. NexCO is calculated by a pulse contour method (CO-TREK) using the measured systolic pressure time integral and the heart's afterload determined from the Windkessel model [16]. 

### 2.3. Measurements

In an 8-hour period, simultaneous CCO and NexCO measurements were obtained every 2 hours (0–2–4–6–8 hrs, in total 225 paired measurements) while simultaneous TDCO and NexCO were obtained every 4 hours (0–4–8 hrs, in total 135 paired measurements). The CCO and NexCO values were recorded simultaneously by hand 5 min before TDCO was determined by 3 repeated injections of 20 mL of sterile ice-cold saline via the central venous line. Blood pressure measurements were recorded continuously by Nexfin and PiCCO and were by each device used to calculate the continuous CO. Subanalysis was performed for patients with a low MAP (defined as MAP ≤ 70 mmHg), low and high TDCO (defined as ≤ 4 L/min and ≥ 8 L/min), low and high SVRI (defined as an SVRI obtained by PiCCO ≤ 1700 dyne·s·cm^−5^/m² and ≥ 3000 dyne·s·cm-5/m²), and patients on high dose norepinephrine (≥0.3 *μ*g/kg/min) and hypothermia (*T*°C ≤ 35°C). To assess the ease of use, we measured the time to initial measurement, the number of repositions needed in the 8-hours observation period and also the nurses filled in a questionnaire (see addendum) (*n* = 27 patients).

### 2.4. Data Analysis and Statistics

Results are presented as mean (±SD) unless otherwise stated. Mean values were compared using student's *t*-test. Paired CO measurements by 2 different methods were compared statistically using 4 different methods. First, we used Pearson correlation and linear regression analysis. Two methods are considered equal if the line of identity crosses the origin of *x* and *y*-axis and if *R* ^2^ (*R* = Pearson's correlation coefficient) is > 0.6. Second, we calculated bias, precision and limits of agreement (Bland-Altman analysis [17, 18]), and the percentage error (PE, defined as two times SD of the bias over the mean TDCO or CCO) as described by L. A. H. Critchley and J. A. J. H. Critchley [19]. If the differences within bias ±1.96 SD (limits of agreement, LA) are not clinically important, if the precision of the new technique is comparable to the reference technique and if the percentage error is less than 30%, the two methods may be used interchangeably [20]. Third, the ability of NexCO to track changes or trends in TDCO or CCO was assessed by plotting ΔTDCO or ΔCCO against ΔNexCO during the same time interval (four quadrants trend plot). The concordance is calculated as the percentage of pairs with the same direction of change. Based on previous reports, the concordance should be > 90% when pairs with both a ΔTDCO or ΔCCO and ΔNexCO ≤ ±1 L/min (or less than 15% of change) are excluded for analysis [21]. Finally trending capability of the NexCO compared to TDCO and CCO was assessed by polar plots as suggested by Critchley et al. [22]. Concordance analysis looking at direction of changes is a very simple but crude measure of how well 2 measurements trend. Important aspects of the measurement, such as the magnitude of the underlying CO change (ΔCO) and the degree of agreement, are totally ignored. Therefore, Critchley et al. suggested converting the *x*-*y* values to polar coordinates, where agreement is shown by the angle the vector makes with the line of identity (*y* = *x*) and magnitude of change by the length of the vector [22]. Thus, statistical measures that fully represent the magnitude of ΔCO and its degree of agreement are retained. From these data, a new polar plot that shows agreement as the angle ***θ*** (angle made by ΔCO vector with the line of identity [*y* = *x*]) against the change in CO as the radian (distance of data point from center of polar plot) can be drawn. Conversion of the Cartesian data with regard to change in cardiac output (ΔCO) into a (*x*, *y*) polar coordinate format was performed using an Excel spreadsheet (Microsoft Office Excel 2007; Microsoft Corp.), with the formulas for calculation of absolute value of mean ΔCO, quadrant, radians, and angle as suggested by Critchley et al. [22].

### 2.5. Ethics

The study was conducted in accordance with the ICU protocol, the declaration of Helsinki, and applicable regulatory requirements as approved by the institutional review board and the local institutional ethics committee (approval number 3789). In view of the nature of the study being purely observational, not demanding a deviation from standard clinical ICU care and since the results obtained by Nexfin were not used for clinical decision making, informed consent from the patient or the next of kin was not deemed essential. We merely analysed the existing situation and did nothing to influence events. Only treating ICU physicians accessed the medical records. All data were pseudonymized before analysis.

## 3. Results

### 3.1. Study Population

In 2 patients (4.3%) it was impossible to obtain Nexfin values from any of 10 fingers, and they were therefore excluded from final analysis. Baseline characteristics of the 45 remaining patients are summarized in Table 1. Thirty-one patients (69%) were in shock (reflected by an elevated arterial lactate) with the majority in septic shock (*n* = 18, 58%). Only a minority was in cardiogenic shock (*n* = 6, 19%). A total of 35 patients (78%) received norepinephrine at a mean (±SD) dose of 0.20 ± 0.17 (range 0.02–1) *μ*g/kg/min, while 27 patients (60%) received dobutamine at a dose of 4.30 ± 2.10 (range 1–10) *μ*g/kg/min (range 1–10). Forty-tree patients (96%) were mechanically ventilated, and the 2 remaining patients were noninvasively ventilated. The critical illness of the patient sample is reflected by high scores on 3 different ICU scoring systems (APACHE II, SOFA, and SAPS II). In-hospital mortality was 57.8%. The neurological, respiratory and hemodynamic parameters, and the dose of the infused drugs did not show significant changes during the entire study period (Table 2).

### 3.2. Cardiac Output

**Figure 1:**
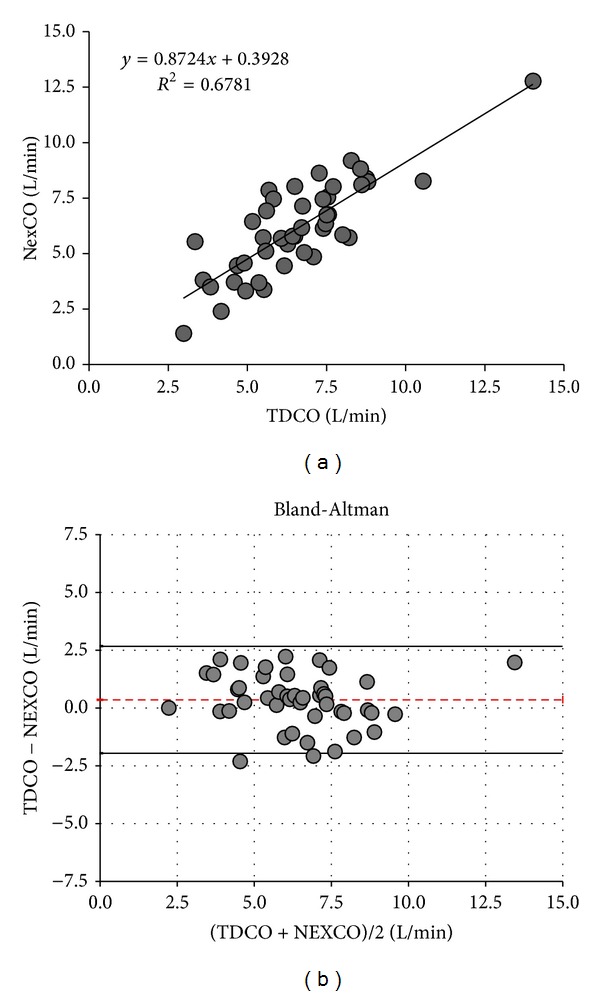
Cardiac output measurements: TDCO versus NexCO. Only one average value per patient is plotted. (a) Regression analysis. (b) Bland-Altman analysis. Patient averages with the mean cardiac output ranges (*x*-axis) and bias errors (*y*-axis) during the 8-hour study period. Dotted line indicates bias and solid lines indicate lower and upper limit of agreement. NexCO: Nexfin cardiac output. TDCO: thermodilution cardiac output.

**Figure 2:**
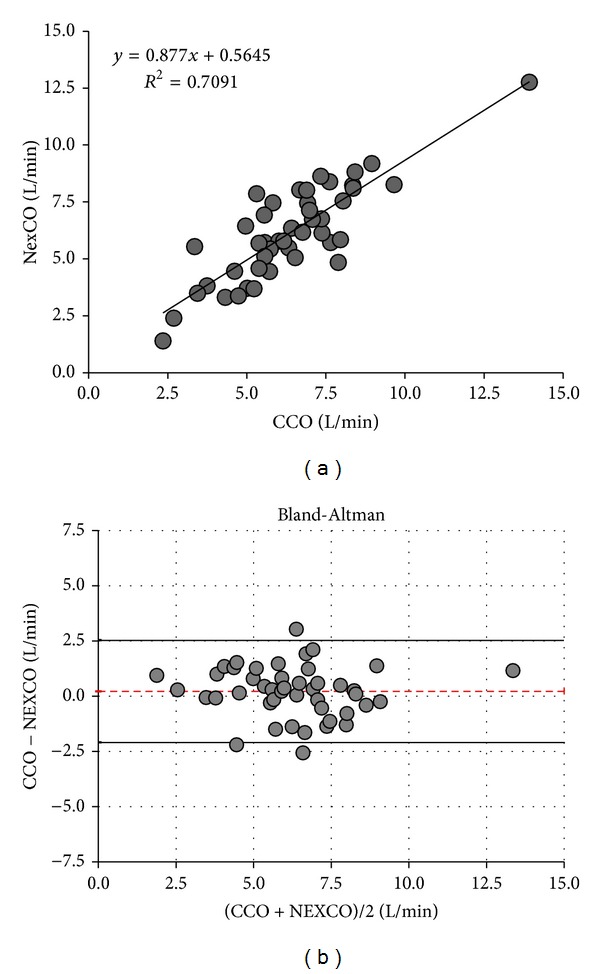
Cardiac output measurements: CCO versus NexCO. Only one average value per patient is plotted. (a) Regression analysis. (b) Bland-Altman analysis. Patient averages with the mean cardiac output ranges (*x*-axis) and errors (*y*-axis) during the 8-hour study period. Dotted line indicates bias and solid lines indicate lower and upper limit of agreement. CCO: pulse contour continuous cardiac output NexCO: Nexfin cardiac output.

Mean NexCO was comparable to mean TDCO (6.1 ± 2.3 versus 6.6 ± 2.2 L/min, *P* = 0.10) and to mean CCO (6.1 ± 2.3 versus 6.4 ± 2.3 L/min, *P* = 0.30). Correlation, regression, and the Bland and Altman analysis are shown in Tables 3 and 4 and Figures 1 and 2. Pearson correlation coefficients comparing NexCO with TDCO (*R* ^2^ 0.68) and NexCO with CCO (*R* ^2^ 0.71) were comparable and showed a highly significant (both *P* values <0.001) correlation between all obtained CO measurements. Bland and Altman analysis comparing NexCO with TDCO revealed a mean bias ±LA of 0.4 ± 2.32 L/min (with 36% error) while analysis comparing NexCO with CCO showed a bias (±LA) of 0.2 ± 2.32 L/min (37% error). TDCO was highly correlated with CCO (*R* ^2^ 0.95, *P* < 0.001) with a bias (±LA) of 0.2 ± 0.86 L/min (13.3% error). 

Subanalysis for patients with a low MAP, high TDCO, low SVRI, and high dose norepinephrine consistently showed a very good correlation between NexCO and TDCO or CCO. NexCO was less reliable in patients with hypothermia and not reliable in patients with low TDCO and high SVRI (Tables 3 and 4).

**Figure 3:**
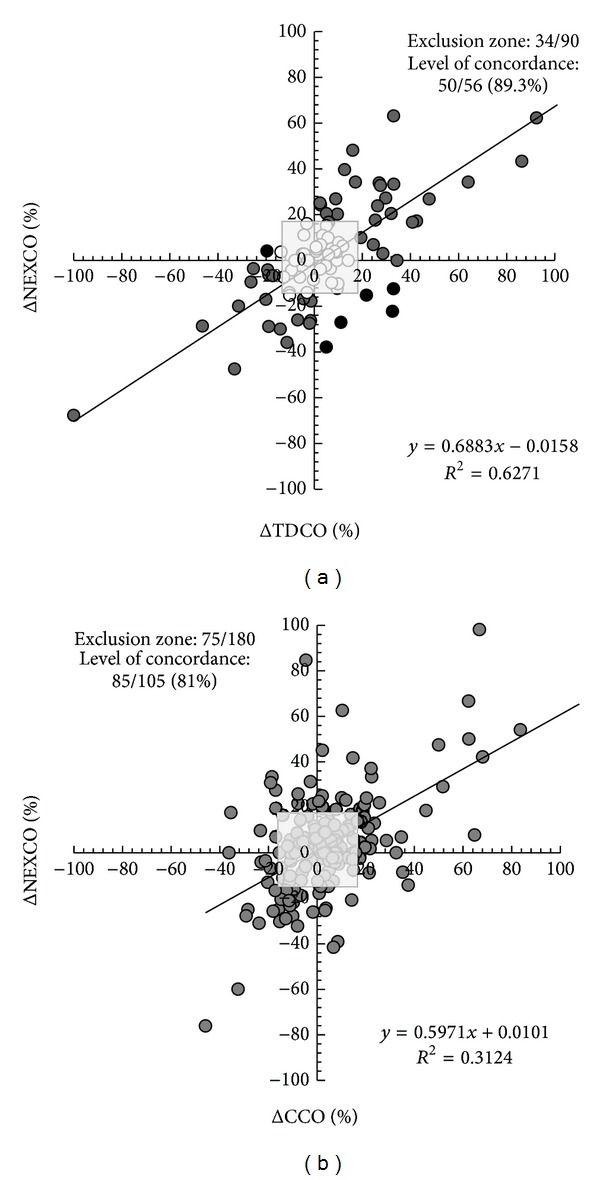
Four quadrants trend plot. (a) Plot for 90 paired measurements of ΔNeXCO and ΔTDCO. From the 90 initial paired measurements, 34 pairs were excluded (exclusion zone is indicated as grey dots within grey-shaded square) because either ΔNexCO or ΔTDCO was ≤ ±15% or because ΔNexCO or ΔTDCO was equal to zero. The calculated level of concordance was 89.3% (50/56) (6 pairs felt within the upper left or lower right quadrant and correspond to poor concordance, black dots). See text for explanation. (b) Plot for 180 paired measurements of ΔNeXCO and ΔCCO. From the 180 initial paired measurements, 75 pairs were excluded (exclusion zone is indicated as grey-shaded square) because either ΔNexCO or ΔCCO was ≤ ±15% change or because ΔNexCO or ΔCCO was equal to zero. The calculated level of concordance was 81% (85/105). See text for explanation.

The four quadrants concordance plots are shown in Figure 3. From the 90 initial paired ΔNexCO/ΔTDCO measurements, 34 pairs was excluded because either ΔNexCO or ΔTDCO was ≤±1 L/min (or ≤15% change) or because ΔNexCO or ΔTDCO were equal to zero (panel (a)). The calculated level of concordance was 89.3% (50/56). The absolute amplitude correlation of these changes was clinically sufficient (*R* ^2^ 0.63, *P* < 0.001). From the 180 initial paired ΔNexCO/ΔCCO measurements, 75 pairs were excluded because either ΔNexCO or ΔCCO was ≤±1 L/min (or ≤15% change) or because ΔNexCO or ΔCCO was equal to zero (panel (b)). The calculated level of concordance was only 81% (85/105). The absolute amplitude correlation of these changes was clinically insufficient but still significant (*R* ^2^ 0.31, *P* = 0.006).

**Figure 4:**
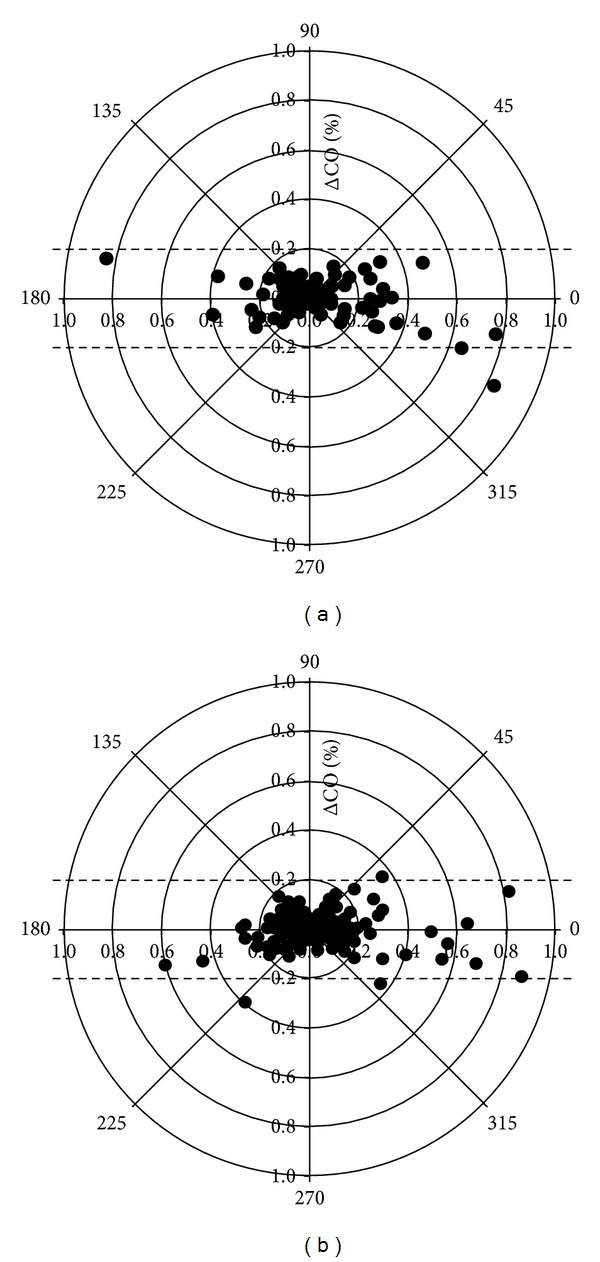
Polar plot. The distance from the center of the plot represents the mean change in cardiac output (ΔCO, expressed as %, with 1,0 referring to 100% change from baseline) and the angle ***θ*** with the horizontal (0-degree radial) axis represents agreement. The less the disagreement between CO measurements, the closer data pairs will lie along the horizontal radial axis. Data with good trending will lie within 10% limits of agreement. However, data with poor trending will be scattered throughout the plot and lie outside the limits of good and acceptable agreement (i.e., 10% and 20%, resp.). See text for explanation. (a) Polar plot for 90 paired measurements of mean ΔCO (%), calculated as absolute value of (ΔNeXCO + ΔTDCO)/2. From the 90 initial data 98.9% of the data points lie within the 20% lines and 89% within the 10% lines, suggesting acceptable trending capabilities. (b) Polar plot for 180 paired measurements of mean ΔCO (%), calculated as absolute value of (ΔNeXCO + ΔCCO)/2. From the 180 initial data 98.3% of the data points lie within the 20% lines and 88.9% within the 10% lines, suggesting acceptable trending capabilities.

The polar trending plots are shown in Figure 4. From the 90 initial data 98.9% of the data points were within the 20% lines and 89% within the 10% lines, suggesting acceptable trending capabilities (Figure 4(a)). From the 180 initial data 98.3% of the data points were within the 20% lines and 88.9% within the 10% lines, suggesting acceptable trending capabilities (Figure 4(b)).

### 3.3. Ease of Use

Data on ease of use were collected in 27 patients. There were no local signs of disturbed circulation in the middle finger due to the application of the finger cuff. The time between the decision to apply Nexfin and the first measurement was less than 5 minutes in 23/27 patients (85%) and between 5 and 10 minutes in 4/27 patients (15%). In 9/27 patients (33%) we were able to do measurements with the first application, while 13/27 patients (48%) needed 1–5 and 5/27 (19%) needed more than 5 repositions. Nurse questionnaires revealed a mean score of 1.4 ± 0.5 for the set-up of the device, 1.7 ± 0.7 for set-up placement, 1.8 ± 0.5 for measurements, and 1.9 ± 0.5 for ease of use (1 = very easy to 4 = very difficult).

## 4. Discussion

We performed an open observational study in 45 mixed surgical/medical and burns critically ill patients to validate the Nexfin against transpulmonary thermodilution and continuous femoral arterial pulse contour derived CO by the PiCCO. To the best of our knowledge, this is the second Nexfin CO validation study conducted in mainly medical ICU patients, the first being published last year by Monnet and coworkers [23]. 

First, we found moderate to good CO correlation coefficients with TDCO (*R* ^2^ 0.68) and CCO (*R* ^2^ 0.71). The overall calculated PEs were however too high to meet the criteria for general interchangeability as suggested by Critchley et al. [22]. These results are in line with previous Nexfin CO validation studies against TEE and PiCCO during abdominal and cardiac surgery [24, 25] and against PAC in a small sample of 10 postsurgical ICU patients [14]. In 2 studies a PE < 30% was reported [14, 25]. Our PE in critically ill patients was lower than the one reported previously by Monnet et al. who found an unacceptable PE of 51% [23]. Second, we found that Nexfin is most accurate in the subgroup of patients with a high CO and low SVRI; however, it was least accurate in patients with low CO and high SVRI. In contrast to other uncalibrated monitoring devices, NexCO keeps comparable reliability in unstable patients with severe hypotension and in patients with reduced vessel compliance due to high dose norepinephrine. In septic patients with the well-known inverse TDCO/SVR hemodynamic profile, with severe hypotension or on high dose norepinephrine the calculated PE was below 30% and all criteria for interchangeability with CCO were met. Since determination of CO was only possible with invasive monitoring in the past, CO is not a target for goal directed therapy guidelines for septic patients [26]. However, since tissue perfusion and oxygen delivery are determined directly by CO and only indirectly by MAP, we strongly believe that with the development and future fine-tuning of noninvasive CO measurement devices like Nexfin, determination of target therapy guidelines for CO should be considered [27]. Third, we found an acceptable concordance (89.3%) between the direction of changes in TDCO and NexCO during the same time interval. This was also shown in previous studies [22, 26]. Although this analysis is based on only 3 time points and 2 values of ΔTDCO/ΔNexCO within an 8-hour time interval, this might be an indication that real-time measurement of the hemodynamic effects of ongoing therapeutic interventions may be reliable with the Nexfin device. Since the absolute ΔTDCO/ΔNexCO amplitude did not show sufficient clinical correlation, clinical decision making should be based on hemodynamic trends rather than on absolute values of changes in measured CO. However, ideal concordance should be above 90%, which was not the case when looking at changes in CCO and NexCO during the same time interval (4 values of ΔCCO/ΔNexCO within each 8-hour interval) with a concordance of only 81%. Fourth, analysis with polar plots showed an acceptable trending capability with 89% of the data points lying within the ±1.0 L/min (or ±10%) limits of agreement lines. Our study is the first to use polar plot analysis in this setting. Finally, the short time to first measurement, the limited number of repositions needed to start measuring, and the high scores on the nurse questionnaires illustrate that the device is very easy to use (“plug and play”) in the majority of patients. However to play the devil's advocate, one could also state that we were unable to use the device in 2 out of 47 patients (4.3%). Monnet et al. found worse results with the inability to obtain CO values in 15.6% of study patients [23].

Based on these results and review of the literature, we think that the Nexfin device can be applied in ICU or ER patients, potentially also on the regular wards and even out of hospital (if the manufacturer would provide a battery), for an initial quick hemodynamic assessment as a bridge to installation of a more advanced invasive monitoring system. Differentiation of the different types of shock on a clinical basis showed to be a major challenge and often inaccurate even in hands of experienced ICU and ER physicians [18]. Also, Nexfin can be applied when catheter placement is problematic for instance in patients with active catheter infections after removal of the previously infected arterial line. 

However, in our opinion there are 5 main reasons why Nexfin cannot always be used as a first choice in the general ICU population with good IV and IA access requiring prolonged advanced hemodynamic monitoring. First, not only is the overall calculated PE too high but we also think that the LA are too broad to be clinically acceptable. If a CO is measured at 8 L/min, the true value can be between 5.7 and 10.3 L/min. Of note; however, is that the obtained correlation coefficients and LA are comparable to previous validation studies with PiCCO against PAC [6–11] and better than results obtained with other noncalibrated, more invasive monitoring devices such as the Vigileo [13, 28], NiCO [13, 29], and PrAM [30]. Also, in a recent meta-analysis, none of the four tested methods achieved satisfactory agreement with bolus thermodilution within the expected 30% PE limits [31]. Therefore, questions are raised on the feasibility of the current validation criteria for uncalibrated CO devices. Second, in our study, Nexfin showed to be less reliable in patients with hypothermia and to be completely unreliable in patients with low TDCO and high SVRI (e.g., cardiogenic, obstructive, and hypovolemic shock). In this subgroup NexCO showed systematic overestimation of CO. This is in line with a previous study conducted in postcardiac surgery ICU patients showing a PE of 50%, mainly driven by inaccuracy in patients with a low CI [32]. This is in contrast however with another study in patients during CABG where half of the patients had a CI < 2.5 L/min/m² and good CO correlation coefficients and PE were still found [25]. Third, Nexfin cannot entirely replace (less) invasive monitoring with an arterial line since arterial blood gas analyses and followup of lactate will always be one of the cornerstones of critical care management. Fourth, in some unstable patients and especially those with changing conditions of preload, afterload, or contractility, it may be advisable to calibrate the CO device in relation to the new hemodynamic situation [33]. Finally, we could not obtain any measurements in 4.3% of patients and others found that the Nexfin could not record the arterial curve due to finger hypoperfusion in 15.6% of patients [23].

Some limitations of this study need to be considered. First, this is a validation study of CO by Nexfin against PiCCO. PAC is still considered by some clinicians as a golden standard. Although highly validated and widely used, PiCCO obtained CO shows some error against PAC. Second, the patient sample size and the size of the subgroups are probably too small to allow extensive further subgroup analysis. Third, we did not perform therapeutic intervention to assess the trending capabilities of the Nexfin. Fourth, we need to be aware that these results were obtained in an ICU patient group already receiving a lot of vasopressors and inotropic hemodynamic support thereby possibly not representing the initial hemodynamic pattern. Future studies should be performed to confirm that these results can be extrapolated to ER patients. Finally, the number of patients was determined on a random basis and no power analysis was performed.

## 5. Conclusions

In conclusion, Nexfin is a totally noninvasive, easy to use blood pressure and CO monitor based on finger arterial blood pressure pulse contour analysis. Nexfin obtained CO showed a moderate to good correlation with CO measured by PiCCO although the PE was too high. The Nexfin can be used for keeping track of changes in CO over time (e.g., to assess the therapeutic effect of a given treatment), although the absolute criteria for full interchangeability were not met in this population of mixed ICU patients.

## Key Messages

- Nexfin is a totally noninvasive, easy to use blood pressure and cardiac output monitor.
- Nexfin shows a moderate to good cardiac output correlation with transcardiopulmonary thermodilution (TDCO) and continuous pulse contour CO (CCO) obtained by PiCCO in a mixed ICU population although the obtained percentage error was too high to allow full interchangeability.
- Changes in NexCO correlate well with changes in TDCO and CCO although the obtained concordance coefficient was too high to allow full trending interchangeability.

## Figures and Tables

**Table 1:** Patient characteristics.

	Mean ± SD	*n* = 45 (100%)
Demographics		
Age (yrs)	57.6 ± 19.4	
Male		32 (71%)
Reason of admission		
Medical		27 (60%)
Surgical		9 (20%)
Trauma		5 (11%)
Burns		4 (9%)
Shock		31 (69%)
Septic		18 (58%)
Cardiogenic		6 (19%)
Other		7 (23%)
ICU scores		
APACHE II	25.3 ± 10.3	
SOFA	9.4 ± 3.3	
SAPS II	51.5 ± 16.9	

APACHE II: acute physiology and chronic health evaluation.

SAPS II: simplified acute physiology score.

SOFA: sequential organ failure assessment.

**Table 2:** Comparison of several neurological, respiratory, and hemodynamic variables and dose of the used drugs between the start and the end (at 8 hours) of the study period.

	Number (%)	Start	8 hours	*P*-value
Neurological				
Propofol (mg/kg/hr)	35 (71%)	2.3 ± 0.9	2.3 ± 1.0	0.99
Midazolam (mg/kg/hr)	31 (69%)	0.2 ± 0.1	0.2 ± 0.1	0.81
Remifentanil (*µ*g/kg/min)	39 (87%)	0.2 ± 0.1	0.2 ± 0.1	0.80
Cisatracurium (mg/kg/hr)	9 (20%)	0.1 ± 0.1	0.1 ± 0.05	0.77
SAS (1–7)	45 (100%)	1.9 ± 1.1	1.6 ± 1.1	0.28
GCS (3–15)	45 (100%)	4.5 ± 3.1	4.8 ± 3.7	0.67
Respiratory system				
pO2/FIO2	45 (100%)	290 ± 171	274 ± 149	0.64
Minute ventilation (L/min)	45 (100%)	11.3 ± 3.5	11.2 ± 4.0	0.92
pH	45 (100%)	7.3 ± 0.1	7.3 ± 0.1	0.45
EVLWI (mL/kg)	45 (100%)	10.2 ± 3.7	10.3 ± 3.4	0.89
Hemodynamics				
Norepinephrine (*µ*g/kg/min)	35 (78%)	0.2 ± 0.2	0.2 ± 0.2	0.90
Dobutamine (*µ*g/kg/min)	27 (60%)	4.1 ± 1.8	4.5 ± 2.2	0.49
MAP (mmHg)	45 (100%)	79.6 ± 16.9	82.9 ± 21.1	0.41
Heart rate (BPM)	45 (100%)	90.3 ± 25.4	88.0 ± 23.7	0.66
CVP (mmHg)	45 (100%)	10.5 ± 5.3	10.1 ± 3.7	0.65
TDCO (L/min)	45 (100%)	6.2 ± 2.1	6.9 ± 2.2	0.14
GEF (%)	45 (100%)	22.8 ± 8.2	24.2 ± 8.6	0.45
GEDVI (mL/BSA)	45 (100%)	715 ± 192	737 ± 162	0.56
SVRI (dyne·s·cm^−5^/m²)	45 (100%)	1868 ± 764	1877 ± 793	0.56
SVV (%)	45 (100%)	15.0 ± 8.8	12.3 ± 7.3	0.12
PPV (%)	45 (100%)	14.6 ± 9.7	12.1 ± 7.9	0.17
Other				
IAP (mmHg)	45 (100%)	7.9 ± 2.9	8.8 ± 3.4	0.15
Body Temperature (°C)	45 (100%)	35.4 ± 1.7	35.5 ± 1.7	0.76

CVP: central venous pressure.

EVLWI: extravascular lung water index.

GCS: Glasgow coma scale.

GEDVI: global end-diastolic volume index.

GEF: global ejection fraction.

IAP: intra-abdominal pressure.

MAP: mean arterial pressure.

PPV: pulse pressure variation.

SAS: sedation and agitation scale.

SVRI: systemic vascular resistance index.

SVV: stroke volume variation.

TDCO: thermodilution cardiac output.

**Table 3:** Comparison of TDCO versus NexCO including subgroup analysis for patients with low MAP, low and high TDCO, low and high SVRI, and high dose norepinephrine and hypothermia.

		Overall	Low MAP	Low TDCO	High TDCO	Low SVRI	High SVRI	Norepinephrine	Hypothermia
			≤70 mmHg	≤4 L/min	≥8 L/min	≤1700 dyne·s·cm^−5^/m²	≥3000 dyne·s·cm^−5^/m²	≥0,3 *µ*g/kg/min	≤35°C
No. of patients		45	19	9	18	29	6	11	22
No. of paired measurements		135	27	16	32	58	14	27	63

Mean TDCO	L/min	6.6	6.2	3.8	8.2	7.2	4.5	6.7	6.5

*R* ^2^		0.67	0.82	0.01	0.67	0.74	0.81	0.89	0.70
*P* value		<0.001	<0.001	NS	<0.001	<0.001	<0.001	<0.001	<0.001

Bias	L/min	0.4	0.9	−0.4	0.9	0.6	−0.1	0.4	0.1
Precision	L/min	1.2	1.1	1.5	1.1	1.3	1.3	1	1.3
Lower LA	L/min	−1.9	−1.2	−3.2	−1.3	−2	−2.7	−1.5	−2.4
Upper LA	L/min	2.7	3	2.5	3	3.2	2.4	2.3	2.6

Percentage error	%	36	34	78	26	37	57	29	39

TDCO: thermodilution cardiac output.

*R* ^2^: Pearson correlation coefficient.

LA: limits of agreement.

MAP: mean arterial pressure.

SVRI: systemic vascular resistance index.

**Table 4:** Comparison of CCO versus NexCO including subgroup analysis for patients with low MAP, low and high TDCO, low and high SVRI, and high dose norepinephrine or hypothermia.

		Overall	Low MAP	Low TDCO	High TDCO	Low SVRI	High SVRI	Norepinephrine	Hypothermia
			≤70 mmHg	≤4 L/min	≥8 L/min	≤1700 dyne·s·cm^−5^/m²	≥3000 dyne·s·cm^−5^/m²	≥0.3 *µ*g/kg/min	≤35°C
No. of patients		45	19	9	18	29	6	11	22
No. of paired measurements		135	50	27	51	104	22	44	63

Mean TDCO	L/min	6.4	5.9	3.8	7.8	7.0	4.5	6.4	6.3

*R* ^2^		0.71	0.87	0.1	0.73	0.81	0.78	0.94	0.67
*P* value		<0.001	<0.001	NS	<0.001	<0.001	<0.001	<0.001	<0.001

Bias	L/min	0.2	0.4	0.0	0.6	0.4	0.0	0.0	0.0
Precision	L/min	1.2	0.9	1.4	1.1	1.0	1.2	0.5	1.3
Lower LA	L/min	−2.1	−1.4	−2.7	−1.7	−1.6	−2.4	−1.1	−2.7
Upper LA	L/min	2.5	2.2	2.7	2.8	2.3	2.3	1.0	2.6

Percentage error	%	36	30	71	29	29	53	16	42

TDCO: thermodilution cardiac output.

*R* ^2^: Pearson correlation coefficient.

LA: limits of agreement.

MAP: mean arterial pressure.

SVRI: systemic vascular resistance index.

## Abbreviations

CCO: Continuous cardiac output by PiCCO
TDCO: Thermodilution
NEXCO: Nexfin obtained cardiac output
CVP: Central venous pressure
EVLWI: Extravascular lung water index
GCS: Glasgow coma scale
GEDVI: Global end-diastolic volume index
GEF: Global ejection fraction
IAP: Intra-abdominal pressure
MAP: Mean arterial pressure
PPV: Pulse pressure variation
PE: Percentage error
SAS: Sedation and agitation scale
SVRI: Systemic vascular resistance index
SVV: Stroke volume variation
SAPS II: Simplified acute physiology score
SOFA: Sequential organ failure assessment
APACHE II: Acute physiology and chronic health evaluation.
